# Supplementary material for: Reduction-Responsive Molecularly Imprinted Poly(2-isopropenyl-2-oxazoline) for Controlled Release of Anticancer Agents
Source: Pharmaceutics. 2020 Jun 2;12(6):506. doi: 10.3390/pharmaceutics12060506 (PMC7356239; doi:10.3390/pharmaceutics12060506)
Supplement: Supplementary file 1 [file pharmaceutics-12-00506-s001.pdf]

pharmaceutics

Supplementary Materials: Reduction-Responsive Molecularly Imprinted Poly(2-isopropenyl-2-oxazoline) for Controlled Release of Anticancer Agents

Michał Cegłowski, Valentin Victor Jerca, Florica Adriana Jerca, Richard Hoogenboom

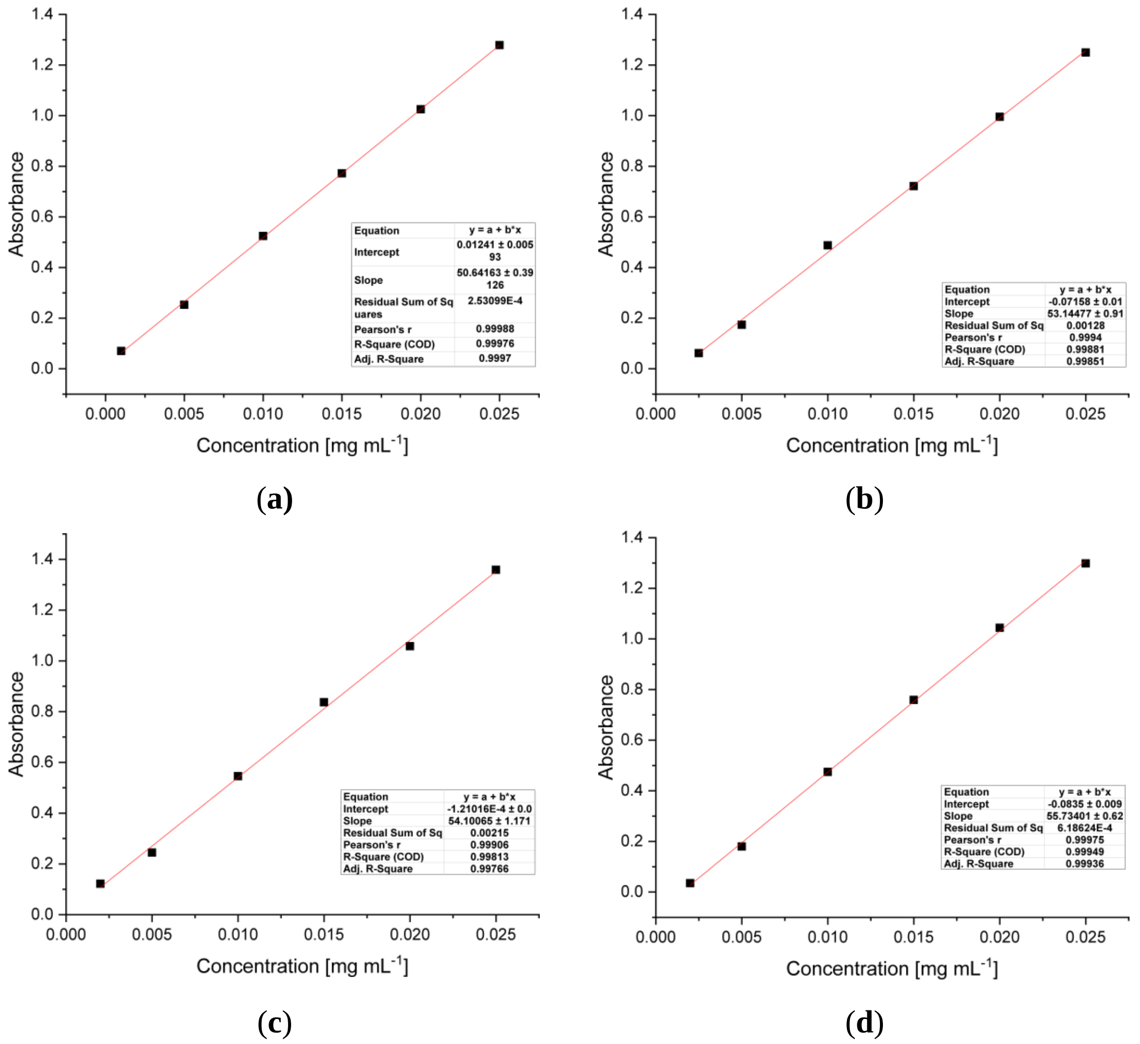

Figure S1. Calibration curves obtained for (**a**) *N*,*N*′-dimethylformamide (DMF) solution; (**b**) pH 7.4; (**c**) pH 6.5; (**d**) pH 2.0.

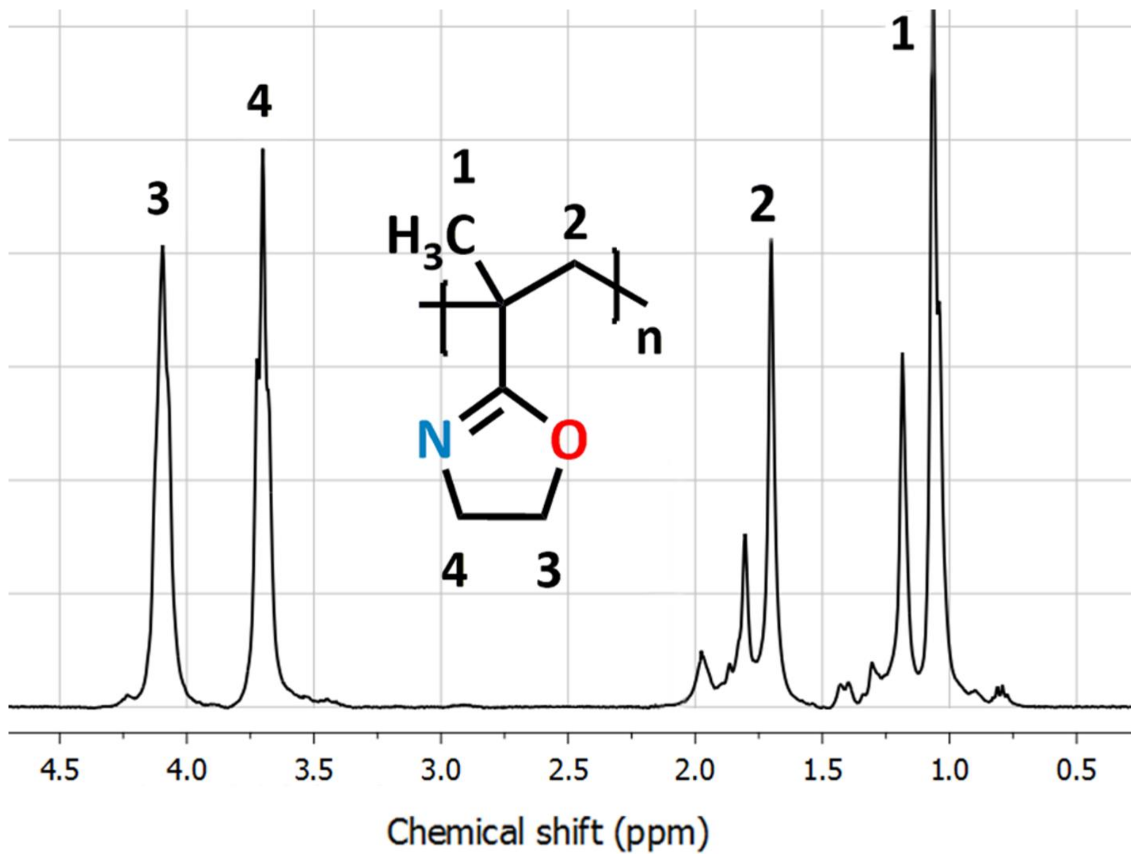

Figure S2. ^{1}H NMR of poly(2-isopropenyl-2-oxazoline) (PiPOx).
